# Supplementary material for: The Influence of Skin Thickness on Flash Glucose Monitoring System Accuracy in Dogs with Diabetes Mellitus
Source: Animals (Basel). 2021 Feb 5;11(2):408. doi: 10.3390/ani11020408 (PMC7914766; doi:10.3390/ani11020408)
Supplement: Supplementary file 1 [file animals-11-00408-s001.pdf]

animals

Table S1: Skin thickness, lifespan of the sensor, removal day of the sensor and day of US examination at T1. NA= not applicable.

	Skin thickness T0 (mm)	Skin thickness T1 (mm)	Sensor Lifespan (days)	Removal day (days from sensor insertion)	US of the neck T1 (day)
**Patient 1***	3.045	3.073	6	7	7
**Patient 2**	3.376	3.328	12	14	14
**Patient 3**	6.303	5.286	10	10	14
**Patient 4**	5.583	5.255	14	14	14
**Patient 5**	5.510	4.950	14	14	14
**Patient 6**	5.858	7.770	14	14	14
**Patient 7**	6.028	NA	2	NA	NA
**Patient 8***	4.675	5.306	13	14	14
**Patient 9**	6.201	7.023	14	14	14
**Patient 10***	4.978	3.995	14	14	14
**Patient 11**	5.591	NA	10	NA	NA
**Patient 12**	3.241	NA	10	NA	NA
**Patient 13**	6.936	7.263	13	14	14
**Patient 14**	2.248	2.015	10	10	14

Copyright: © 2021 by the authors. Submitted for possible open access publication under the terms and conditions of the Creative Commons Attribution (CC BY) license (http://creativecommons.org/licenses/by/4.0/).
